# Visuospatial but Not Verbal Working Memory Deficits in Adult Patients With Neurofibromatosis Type 1

**DOI:** 10.3389/fpsyg.2021.751384

**Published:** 2021-11-11

**Authors:** Hanlu Tang, Qiong Wu, Shiwei Li, Yehong Fang, Zhijun Yang, Bo Wang, Xingchao Wang, Pinan Liu

**Affiliations:** ^1^Department of Neurosurgery, Beijing Tiantan Hospital, Capital Medical University, Beijing, China; ^2^Beijing Key Laboratory of Learning and Cognition, School of Psychology, Capital Normal University, Beijing, China; ^3^China National Clinical Research Center for Neurological Diseases, Beijing, China; ^4^Department of Neural Reconstruction, Beijing Neurosurgery Institute, Capital Medical University, Beijing, China

**Keywords:** neurofibromatosis type 1, working memory, visuospatial, verbal, adult

## Abstract

**Background:** Cognitive dysfunction is one of the main symptoms of neurofibromatosis type 1 (NF1). As an important cognitive function, working memory (WM) has rarely been systematically analyzed in NF1 by isolating the particular domain of WM, and existing data involving WM in adult patients with NF1 are insufficient. This study aimed to investigate the characteristics of different types of WM in NF1 from the perspective of the adult population.

**Method:** We comprehensively analyzed WM in both verbal and visuospatial WM domains by using the N-back task (including the verbal N-back task and the visuospatial N-back task) in 31 adults with NF1 and 34 healthy controls matched for age, gender, education levels, and general cognitive status. The accuracy and reaction times (RTs) in the N-back task were entered into mixed-design ANOVA.

**Results:** Compared with healthy controls, adults with NF1 presented significantly lower mean accuracy and longer RTs in the visuospatial N-back task. However, no significant difference was found between the NF1 group and healthy controls in the verbal N-back task.

**Conclusions:** The present study suggested that adults with NF1 might have deficits in visuospatial WM. We did not find evidence for verbal WM deficits in adult patients with NF1. Our findings supplement and refine the existing data on WM in the context of NF1.

## Introduction

Neurofibromatosis type 1 (NF1) is an autosomal-dominant disorder with an average global prevalence of ~1/3000 (Gutmann et al., 2017). The disease not only causes neurofibromas, café-au-lait macules, optic pathway gliomas, and malignant peripheral nerve sheath tumors but also leads to structural changes in the brain (Duarte et al., 2014; Gutmann et al., 2017) as well as various cognitive dysfunctions (Torres Nupan et al., 2017). Compared with unaffected peers, individuals with NF1 usually exhibit deficits in many important cognitive domains, such as IQ (Lehtonen et al., 2015), visual perception (Bulgheroni et al., 2019), language (Cosyns et al., 2012), reading (Torres Nupan et al., 2017), calculation (Burgio et al., 2017), attention (Lehtonen et al., 2013; Wang et al., 2019), and executive function (Beaussart et al., 2018). In addition, working memory (WM), an important cognitive function, has received increasing attention from clinicians.

WM is an important part of cognitive processing, providing temporary storage and manipulating essential information for complex cognitive activities (Baddeley and Hitch, 1974; Eriksson et al., 2015). Based on the most influential model of WM provided by Baddeley, WM can be subdivided into verbal WM and visuospatial WM (Baddeley, 2012), involving the temporary maintenance and manipulation of verbal and visuospatial information, respectively (Acheson and MacDonald, 2009; McAfoose and Baune, 2009). Their deficits will exert important effects on education- and work-related activities and consequently affect the quality of life.

To date, previous NF1 studies involving WM have mainly focused on children (Ferner et al., 1996; Hyman et al., 2005; Rowbotham et al., 2009; Huijbregts et al., 2010; Ullrich et al., 2010; Payne et al., 2011, 2012; Lorenzo et al., 2013; Champion et al., 2014; Gilboa et al., 2014; Lehtonen et al., 2015; Plasschaert et al., 2016; Casnar and Klein-Tasman, 2017; Chaix et al., 2017), with few data involving adult populations (Shilyansky et al., 2010; Descheemaeker et al., 2013), which limits our further understanding of NF1 disease from the perspective of population characteristics. We need more data on adults with NF1 to explore the features of WM in NF1 patients across the lifespan. Moreover, most prior NF1 studies focused on the general estimation of WM or paid only one-sided attention to a certain type of WM. In addition, some of the patterns observed across studies have not been consistent. Rowbotham et al. (2009) and Gilboa et al. (2014) found that NF1 children presented poorer WM than matched healthy peers. Shilyansky et al. (2010) observed that adults with NF1 presented poorer spatial WM than healthy controls. Hyman et al. (2005) revealed that there was no significant difference between NF1 children and matched peers in verbal WM. Descheemaeker et al. (2013) reported auditory WM deficits in adults with NF1. Chaix et al. (2017) discovered no auditory-verbal WM and phonological short-term WM impairment in children with NF1. Few studies have conducted an integrated analysis of different types of WM simultaneously by isolating different domains of WM.

To address the previous problems and biases, the current study recruited adults with NF1 and matched healthy controls to comprehensively analyze the features of WM in individuals with NF1 in both verbal and visuospatial WM domains based on Baddeley's WM model (Baddeley, 2012) by using the N-back task (Kane et al., 2007), which allows the verbal and visuospatial domains of WM to be precisely and simultaneously examined. The aims of our study were as follows: (1) to determine whether adults with NF1 exhibit deficits in WM (including verbal and visuospatial WM) and, if so, to clarify the characteristics of the deficits; and (2) to supplement and refine the existing data on WM in NF1 disease to provide a theoretical basis for clinical drug therapy and psychological intervention. Based on data from previous studies on children and adolescents with NF1 (see Supplementary Table 1), we hypothesized that NF1 adults would present deficits in visuospatial WM and no deficits in verbal WM.

## Materials and Methods

### Participants

We recruited 33 adult patients with NF1 from the Neurofibromatosis Outpatient Department of Beijing Tiantan Hospital between 2019 and 2020. Thirty-six healthy controls were recruited from the community. Two patients were excluded for not completing the task, and two healthy controls were excluded due to misunderstanding the instructions before the task. The final sample included 31 adults with NF1 and 34 healthy controls. All individuals with NF1 fulfilled the diagnostic criteria established by the National Institutes of Health Consensus Development Conference (Stumpf, 1988), and most of them had café-au-lait macules or small benign subcutaneous nodules on the body, but these skin lesions did not seriously affect their basic daily activities. All patients were clinically stable, and none of them presented abnormalities on general neurological examination or limitations in daily life. All participants were right-handed and had normal or corrected-to-normal vision.

All participants were required to complete the short form of the Beck Depression Inventory (BDI-SF) (Beck and Beck, 1972) and the Mini-Mental State Examination (MMSE) (Folstein et al., 1975). The BDI-SF was used to quantify the general emotional state of the participants to avoid interference from emotional factors. Scores below or equal to 4 points indicate no or minimal depressive symptoms. Scores above this threshold indicate mild (5–7 points), moderate (8–15 points), or severe (≥16 points) depressive symptoms. The MMSE was used to assess the general cognitive status of the participants to ensure their ability to understand and cooperate in the further tasks of advanced cognitive function. Any score greater than or equal to 25 points (out of 30) indicates normal cognition. Scores below this threshold indicate mild (21–24 points), moderate (10–20 points), or severe (≤ 9 points) cognitive deficits.

The admission criteria for the individuals with NF1 and the healthy controls were (1) 18 ≤ age ≤ 60 y; (2) an MMSE score ≥ 24; (3) no severe NF1 symptoms, such as plexiform neurofibromas or malignant peripheral nerve sheath tumors that cause pain and may have psychological effects; (4) no intracranial surgery history; (5) no history of serious chronic disease; (6) no psychiatric disorders and family history of psychiatric disorders; (7) no recent use of any medications that could affect cognitive abilities; and (8) voluntary participation as given by a signed consent document. All participants experienced the same experimental procedures in the same quiet room. All tests were administered by the same tester. This study was approved by the Medical Ethics Committee of Beijing Tiantan Hospital, Capital Medical University, China.

The final sample included 31 adults with NF1 (12 males and 19 females) and 34 healthy controls (10 males and 24 females). The ages of the individuals with NF1 and the healthy controls were 30.4 ± 7.7 and 31.1 ± 9.8 y, respectively. The years of education of the individuals with NF1 and the healthy controls were 12.8 ± 3.1 and 13.0 ± 3.3 y, respectively. No individuals with NF1 or healthy controls presented with cognitive deficits as measured by the MMSE (28.8 ± 1.2 and 29.2 ± 0.9, respectively). The BDI-SF scores of the individuals with NF1 indicated moderate depressive mood, and the BDI-SF scores of the healthy controls indicated no or minimal depressive mood (8.9 ± 7.5 and 2.6 ± 3.5, respectively). The individuals with NF1 and healthy controls were matched for age [*t*_(63)_ = −0.33, *p* = 0.741], gender [$\chi_{\left(63\right)}^{2}$ = 0.63, *p* = 0.429], and educational attainment [*t*_(63)_ = −0.24, *p* = 0.810]. There was no significant difference in MMSE score between NF1 patients and healthy controls [*t'*_(63)_ = −1.65, *p* = 0.105]. The BDI-SF score [*t'*_(63)_ = 4.28, *p* < 0.001] was not matched and was removed as a covariate in the data analysis (Table 1).

**Table 1:** Demographic characteristics of participants.

	**Age (years)**	**Sex**	**Education (years)**	**MMSE**	**BDI-SF**
	**Mean (95%CI)**		**Mean (95% CI)**	**Mean (95% CI)**	**Mean (95% CI)**
NF1	30.4 (2.8)	12 males	12.8 (1.1)	28.8 (0.4)	8.9 (2.7)
HCs	31.1 (3.4)	10 males	13.0 (1.2)	29.2 (0.3)	2.6 (1.2)
*p*-value	0.741	0.429	0.810	0.105	<0.001

*Age, the age at the testing date; BDI, the short form of the Beck Depression Inventory, a measure of baseline mood; CI, confidence interval; HCs, healthy controls; MMSE, Mini-Mental Status Examination, a test for cognitive impairment; NF1, patients with neurofibromatosis type 1*.

### The N-Back Task

The N-back task was used to measure the participants' accuracy and reaction times (RTs) to the stimulus under various memory load levels, including the visuospatial N-back task and the verbal N-back task, which examined the abilities of visuospatial WM and verbal WM, respectively (Callicott et al., 1999; Kane et al., 2007; Chatham et al., 2011; Chen et al., 2019). The participants were required to determine whether the presented stimulus was the same as the Nth stimulus before (Owen et al., 2005). The order of administration of the verbal and visuospatial N-back tasks was fixed between participants. All participants conducted verbal N-back task at first, which was relatively easier and more suitable for subjects to become familiar with the complete N-back task. The N-back program runs on the E-prime™ (Psychology Software Tools, Pittsburgh, PA, USA).

**Figure 1:**
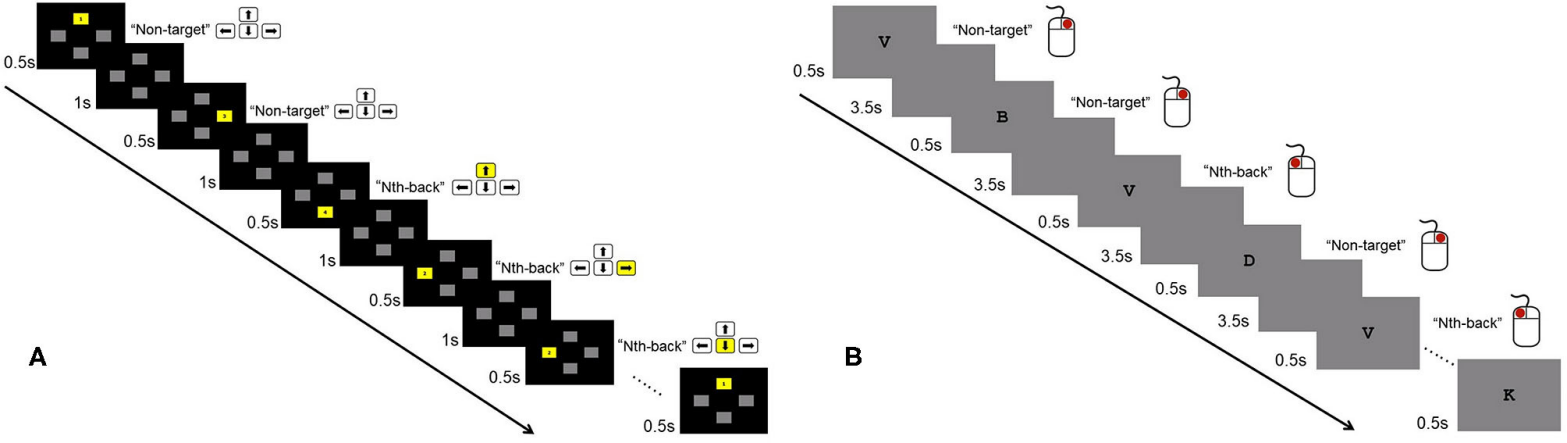
Illustration of the 2-back condition of the visuospatial and verbal N-back tasks. **(A)** In the visuospatial N-back task, participants were instructed to indicate the location of the previous Nth (1- and 2-back) box that turned yellow using the direction keys of left, right, up, and down. For the 0-back condition, participants pressed the corresponding key as the box turned yellow. **(B)** In the verbal N-back task, participants pressed the left button if the current letter was the same as the Nth (1-, 2-, and 3-back) letter before; otherwise, they pressed the right button. For the 0-back condition, participants were required to indicate whether the current letter was “X.”

In the visuospatial N-back task, four gray boxes (up, down, left, right) were located in the center of the screen (Figure 1A). In each trial, a random box turned yellow for 500 ms followed by an interstimulus interval of 1000 ms. There were three conditions: 0-back, 1-back, and 2-back. The participants were instructed to indicate the location of the previous Nth box that turned yellow using the left, right, up, and down direction keys. In the 0-back condition, the participants pressed the corresponding key as the box turned yellow. The participants' RTs and accuracy were recorded. Each condition consisted of six blocks. Eighteen blocks presented in random order. Each block contained 20 trials and lasted ~30 s. The total time of the visuospatial N-back task was ~9 min. The participants practiced the task for 3 blocks (0-back, 1-back, 2-back each) before the formal test began.

In the verbal N-back task, a series of letters were presented one by one in the center of the screen, and each letter was followed by a 3500 ms blank screen (Figure 1B). The four blocks (0-back, 1-back, 2-back, 3-back) were presented once in a fixed order, with 18 letters in each block. In each trial, the participants were asked to press the left button if the current letter was the same as the Nth letter before; otherwise, they pressed the right button. For the 0-back condition, the participants were required to indicate whether the current letter was “X.” The participants' accuracy and RTs were recorded. The total time for the verbal N-back task was ~5 min.

### Data Analysis

The data from visuospatial and verbal N-back tasks were analyzed separately because there were different levels of N in each task. The accuracy and RTs were entered into mixed-design ANOVA, with the task difficulty level (3 for the visuospatial N-back task and 4 for the verbal N-back task) as the within-subject factor and the group (NF1 and healthy controls) as the between-subject factor. Incorrect responses were excluded from the computation of RTs. Since the individuals with NF1 showed a slightly depressed mood, as indicated by higher BDI-SF scores than those of the healthy controls, we controlled this potential confounding factor by regressing out the BDI scores as the covariate.

## Results

**Figure 2:**
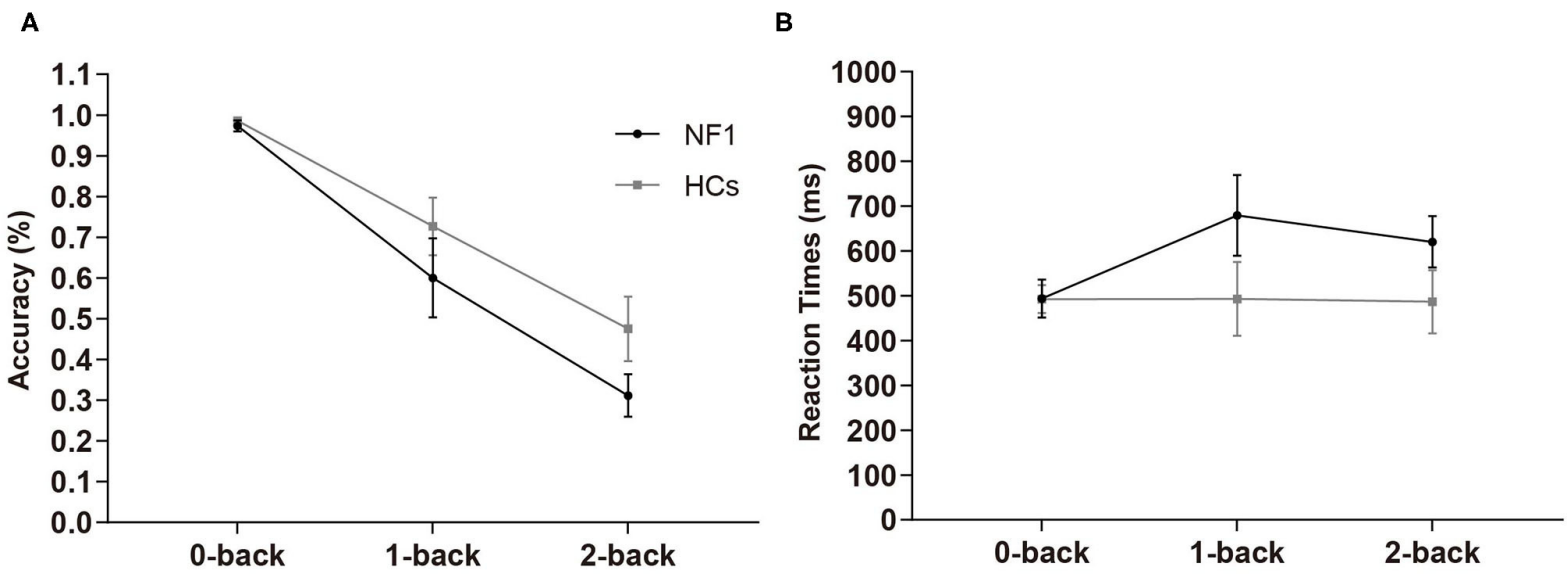
Results of accuracy **(A)** and reaction times **(B)** in the visuospatial N-back task. Adult patients with NF1 performed poorer visuospatial WM than HCs. Error bar was 95% confidence interval. HCs, healthy controls; NF1, neurofibromatosis type 1.

In the visuospatial N-back task, the overall mean accuracies of the NF1 and healthy controls were 62.84% ± 12.67% and 72.94% ± 13.41%, respectively, and their RTs were 598 ± 145 ms and 491 ± 158 ms, respectively (see Table 2 for details). The mixed-design ANOVA for accuracy showed a significant main effect of task difficulty level [*F*_(2, 124)_ = 119.91, *p* < 0.001; Figure 2A], with less accurate responses as the difficulty levels increase. Importantly, we found a significant main effect of group [*F*_(1, 62)_ = 4.60, *p* = 0.036]. Patients with NF1 showed lower performance accuracy than healthy controls, indicating a potential impairment in visuospatial WM. There was no interaction between group and task difficulty [*F*_(2, 124)_ = 2.16, *p* = 0.120]. The mixed-design ANOVA for RTs revealed similar results {task difficulty main effect: [*F*_(2, 124)_ = 3.85, *p* = 0.024]; group main effect: [*F*_(1, 62)_ = 4.91, *p* = 0.030]; Figure 2B}. Patients with NF1 were slower than the healthy controls, which was mainly caused by difficult levels of the task (i.e., 1-back and 2-back), as evidenced by a significant interaction effect on RTs [*F*_(2, 124)_ = 6.50, *p* = 0.002].

**Table 2:** The details of the N-back task.

	**NF1**				**HCs**			
	**Accuracy (%)**		**RTs (ms)**		**Accuracy (%)**		**RTs (ms)**	
	**Mean**	**SD**	**Mean**	**SD**	**Mean**	**SD**	**Mean**	**SD**
**Visuospatial N-back task**								
0-back	97.34%	3.65%	494	115	98.65%	2.06%	493	90
1-back	60.04%	26.46%	679	246	72.66%	20.34%	493	237
2-back	31.16%	14.29%	620	156	47.52%	22.64%	487	202
**Verbal N-back task**								
0-back	94.62%	5.46%	591	120	96.73%	4.95%	610	128
1-back	91.46%	10.06%	728	180	96.19%	5.95%	710	183
2-back	84.07%	14.33%	802	157	90.80%	9.13%	877	255
3-back	78.49%	16.12%	890	268	84.90%	11.41%	976	321

*HCs, health controls; NF1, patients with neurofibromatosis type 1; RTs, reaction times; SD, standard deviation*.

**Figure 3:**
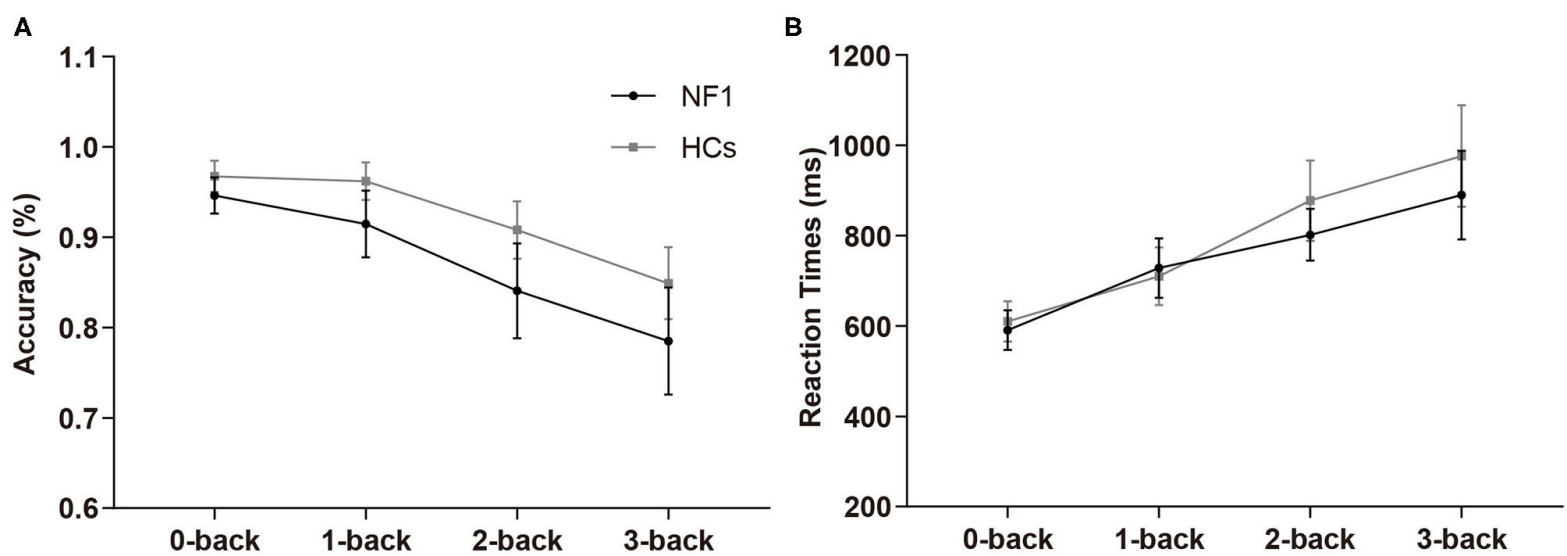
Results of accuracy **(A)** and reaction times **(B)** in the verbal N-back task. No difference was found in verbal WM between adult patients with NF1 and HCs. Error bar was 95% confidence interval. HCs, healthy controls; NF1, neurofibromatosis type 1.

In the verbal N-back task, the overall mean accuracies of the NF1 pateints and healthy controls were 87.16% ± 8.31% and 92.16% ± 4.71%, respectively, and their RTs were 753 ± 157 ms and 793 ± 193 ms, respectively (see Table 2 for details). The mixed design ANOVAs for accuracy and RTs both revealed significant main effects of task difficulty level {accuracy: [*F*_(3, 186)_ = 10.94, *p* < 0.001]; RTs: [*F*_(3, 186)_ = 46.63, *p* < 0.001]; Figures 3A,B}, with less accurate and slower responses as the difficulty levels increased. Interestingly, we did not find a significant main effect of group {accuracy: [*F*_(1, 62)_ = 2.41, *p* = 0.13]; RTs: [*F*_(1, 62)_ = 0.86, *p* = 0.36]} or its interaction with task difficulty for either accuracy or RTs {accuracy: [*F*_(3, 186)_ = 0.87, *p* = 0.46]; RTs: [*F*_(3, 186)_ = 1.01, *p* = 0.36]}, indicating no deficit of verbal WM ability found in the participants with NF1 compared to healthy controls^1^.

Together, the results from the visuospatial and verbal N-back tasks suggest that the adult patients with NF1 may have deficits in visuospatial WM but no evidence of deficits in verbal WM was found in NF1 participants.

## Discussion

In the current study, we analyzed WM in 31 adults with NF1 and 34 healthy controls by using the N-back task. The results showed that adults with NF1 might present deficits in visuospatial WM and the present study did not find a difference in verbal WM between adults with NF1 and healthy controls, which was consistent with our prior hypotheses.

### Potential Visuospatial WM Deficits in Adult Patients With NF1

Visuospatial WM supports perception, attention, actions to guide thought and higher-level cognition (Mance and Vogel, 2013; Thomas, 2013). In the visuospatial N-back task, the results showed that the accuracy decreased as task difficulty levels increased in both NF1 patients and healthy controls. The mean accuracy of NF1 was significantly lower than that of healthy controls. In the 0-back task, there was no difference between adults with NF1 and healthy controls. However, as the task difficulty levels increased, the gap between the NF1 patients and healthy controls gradually became obvious, especially in the 2-back task. The result of RTs revealed a similar phenomenon. NF1 patients spent more time than healthy controls in the whole visuospatial N-back task. The above results suggested that adults with NF1 might exhibit potential deficits in visuospatial WM compared with healthy controls, and the deficits became more obvious as memory load increased. Our finding was consistent with those of Huijbregts et al. (2010), in which WM deficits became apparent in children with NF1 as task difficulty increased.

The deficit of visuospatial WM was also reported in children and adolescents with NF1 (see Supplementary Table 1), which suggested that this deficit could occur early in the life of individuals with NF1 and affect the development and academic achievement of school-age patients. Although a study recruiting five elderly individuals with NF1 (age > 60 y, mean age 65 y) found that elderly NF1 patients presented spatial WM impairments compared with healthy controls (Costa Dde et al., 2014), few previous studies noticed visuospatial WM deficits in adult populations. Shilyansky et al. (2010) recruited 14 adults with NF1 and assessed their spatial WM via two spatial delayed response tasks. Their results showed that adults with NF1 presented significantly lower accuracy than healthy controls, with an apparent decline as memory load increased. In addition, combined with our reviewed previous literature involving WM in individuals with NF1 (see Supplementary Table 1), we found visuospatial WM dysfunction to be a typical feature of NF1 patients across the lifespan (from childhood to advanced adulthood). Therefore, we should pay more attention to WM dysfunction in individuals with NF1 in the clinic.

### No Verbal WM Deficit Was Found in Adult Patients With NF1

Verbal WM supports language such as syntactic and semantic operations, as well as vocabulary acquisition during development (Cogan et al., 2017). The present study did not find a difference in verbal WM between adults with NF1 and healthy controls, which supported two previous studies on pediatric patients. Hyman et al. (2005) found that there was no noticeable difference between NF1 patients and healthy controls in verbal WM, as assessed by both the Digit Span Backwards test and the Digit Span Forwards minus Digit Span Backward test. Chaix et al. (2017) reported no auditory-verbal WM and phonological short-term WM impairment in children with NF1 via the “WM index” of the Wechsler Intelligence Scale for Children–Fourth Edition and the pseudoword repetition task, respectively. Notably, the descriptions and definitions of verbal WM in previous studies varied, lacking universal terminology. Different researchers defined verbal WM differently based on their educational background and professional affiliation, which made it difficult for us to interpret and compare the results of these studies.

However, several previous studies put forward different findings. Descheemaeker et al. (2013) reported auditory WM deficits in adults with NF1 through the auditory verbal learning test (Dutch version). Costa Dde et al. (2014) found that elderly individuals with NF1 presented verbal WM impairments compared with healthy controls through Digit Span Backwards and Digit Span Forward tests. A possible reason for these conflicting results may be that the methods (such as batteries) used to assess verbal WM varied (see Supplementary Table 1). Each measurement method of WM has its own focus. For example, the “WM index” of the Wechsler Adult Intelligence Scale is biased toward a composite score. Digit Span Forwards and Backwards tests essentially measure attention and the executive component of WM, respectively (Kent, 2016). In the current study, we used the N-back task, which can examine verbal and visuospatial WM simultaneously, with the advantage of manipulating memory load by controlling the number of stimuli between the current stimulus and the target stimulus to increase memory load while eliminating other interfering factors (Owen et al., 2005). The N-back paradigm was designed to engage the WM system in rapidly encoding, maintaining, and updating information.

### Potential Neural Mechanisms Underlying WM Dysfunction in NF1

As a monogenic disease, NF1 provides a unique genetic model to explore and mechanistically dissect the molecular mechanism underlying WM. *NF1* gene mutation is the core cause of WM impairment in NF1 patients. Reduced *NF1* gene expression in neurons leads to decreased neurofibromin production, resulting in abnormalities in the downstream molecular and signaling pathway related to memory, such as reduced cyclic adenosine monophosphate levels (Ho et al., 2007; Diggs-Andrews and Gutmann, 2013), increased GABA release (Shilyansky et al., 2010), and reduced long-term potentiation (Diggs-Andrews and Gutmann, 2013). Furthermore, the decline of dopamine (DA) in the brain is associated with WM impairments (Cools and D'Esposito, 2011). Methylphenidate, a DA reuptake inhibitor, can ameliorate WM deficits in children with NF1. Moreover, DA administration rescued the performance of mice in the Morris water maze test (a task measuring spatial WM), and DA D1 receptor agonist treatment corrected the abnormalities of the long-term potentiation in hippocampal slice preparations *in vitro*. Additionally, an animal experiment also found that heterozygous mice with an *NF1* null mutation have reduced DA levels in the hippocampus (Diggs-Andrews et al., 2013). These findings support the opinion that brain DA levels may play a pivotal role in WM deficits in NF1 patients.

Additionally, the hypoactivation of key components of *WM* circuitry (the right parietal cortex and the left dorsolateral prefrontal cortex) and aberrant functional connectivity in individuals with NF1 may underlie their visuospatial WM difficulties (Ibrahim et al., 2017). Furthermore, individuals with NF1 show a more diffuse pattern of increased brain activation than healthy controls during high- vs. low-memory-load tasks, which may reflect a less efficient pattern of brain activity (Ibrahim et al., 2017). This could explain why, in our study, the participants with NF1 performed worse than healthy controls during high-visuospatial-memory-load tasks (1-back and 2-back tasks) but not during low-memory-load tasks (0-back task). NF1 patients also presented visuospatial impairment (Hyman et al., 2005; Rowbotham et al., 2009). Violante et al. (2012) provided fMRI evidence that visuospatial deficits in patients with NF1 could be associated with a dysfunction in the visual cortex, especially in the magnocellular pathway, which might provide a neural explanation for visuospatial WM dysfunction in NF1. Further investigations of the neural mechanisms underlying WM dysfunction in NF1 will be conducted in the future.

### Limitations

This study presented the following limitations: (1) This study was based on a single experiment and should be replicated. (2) Both the NF1 and control groups included participants who varied in age from 18 to 60 years. This large range can potentially exert a large effect on scores because cognitive aging studies have shown significantly better WM performance for younger adults compared to older adults. (3) The N-back task may simultaneously evaluate other cognitive processes besides WM, which may be a disadvantage of this task. (4) The current study evaluated WM by only using the N-back task. Performance on an isolated task could depend on several other factors, including deficits in other cognitive processes, not only WM. As our previous study confirmed that NF1 patients presented selective impairment of the executive attentional network (Wang et al., 2019). We may find out the potential interaction of multiple cognitive functions and their effect on WM in further study. In addition, we will conduct a more comprehensive cognitive assessment of NF1 WM deficits by adding other measurements (e.g., Digit Span, Corsi block task, WM Index of the Wechsler Intelligence Scale, etc.).

## Conclusion

Compared with healthy controls, adults with NF1 may have deficits in visuospatial WM, and visuospatial WM dysfunction becomes more obvious with the increase of memory load. Visuospatial WM dysfunction is a typical feature of NF1 across the lifespan (from childhood to advanced adulthood). No deficit of verbal WM was found in adults with NF1 in the present study. Decreased NF1 gene expression and its downstream molecular and signaling pathway abnormalities as well as local brain neuronal activity abnormalities may be the potential neural mechanisms underlying WM dysfunction in NF1. Our results supplement and refine WM data of NF1 to provide a theoretical basis for clinical drug therapy and psychological intervention.

## Data Availability Statement

The raw data supporting the conclusions of this article will be made available by the authors, without undue reservation.

## Ethics Statement

This study was approved by the Medical Ethics Committee of Beijing Tiantan Hospital, Capital Medical University, China. The patients/participants provided their written informed consent to participate in this study.

## Author Contributions

PL, XW, and QW designed the study. Data collection was performed by HT and SL. HT and QW performed data analysis and wrote the first and successive versions of the manuscript. All authors contributed to the interpretation of the results, intellectual content, critical revisions to the drafts of the paper, and approved the final version.

## Funding

This work was supported by the National Natural Science Foundation of China (grant numbers: 81974387 to PL, 31900755 to QW, 81729001, 81600931 to XW). PL was also supported by the Beijing Municipal Science and Technology Commission (grant number: Z161100002616014).

## Conflict of Interest

The authors declare that the research was conducted in the absence of any commercial or financial relationships that could be construed as a potential conflict of interest.

## Publisher's Note

All claims expressed in this article are solely those of the authors and do not necessarily represent those of their affiliated organizations, or those of the publisher, the editors and the reviewers. Any product that may be evaluated in this article, or claim that may be made by its manufacturer, is not guaranteed or endorsed by the publisher.

## Footnotes

^1^Although the NF1 patient group and healthy controls matched in age, we still tested whether our results held after removing the potential confounding effect of age as a covariate. The visuospatial and verbal WM tasks got similar results compared to previous one, demonstrating an impairment in visuospatial but not in verbal WM.
