# Supplementary material for: Visuospatial but Not Verbal Working Memory Deficits in Adult Patients With Neurofibromatosis Type 1
Source: Front Psychol. 2021 Nov 11;12:751384. doi: 10.3389/fpsyg.2021.751384 (PMC8631787; doi:10.3389/fpsyg.2021.751384)
Supplement: Supplementary file 1 [file Table_1.DOCX]

| Studies | age | Groups | Task | Assessed construct  (as mentioned in the study) | Group differences |
| --- | --- | --- | --- | --- | --- |
| Lorenzo J et al., 2013^[1]^ | NF1 mean age=40.23 mo  NCs mean age=40.16 mo | NF1=43  NCs=43 | The Delay Alternation task | Nonverbal WM | — |
| Casnar C L et al., 2017^[2]^ | NF1 mean age=4.53 y  NCs mean age=4.51 y | NF1=26  NCs=37 | BRIEF-P | WM | NF1 < NCs |
| Chaix Y et al., 2018^[3]^ | NF1 mean age=10 y  NCs mean age=10 y | NF1=75  NCs=75 | Corsi blocks  WMI of the WISC-IV  The pseudoword repetition task | Spatial WM Forward span  Spatial WM Backward span  Auditory-verbal WM  Phonological short-term WM | —  NF1 < NCs  —  — |
| Payne J M et al., 2012^[4]^ | NF1 mean age=11 y  NCs mean age=10 y | NF1=49  NCs=30 | CANTAB | Spatial WM | NF1 < NCs |
| Lehtonen A et al., 2015^[5]^ | NF1 mean age=11y9 mo  NCs mean age=10y7 mo | NF1=49  NCs=19 | Spatial WM task  BRIEF | Spatial WM  WM | NF1 < NCs  NF1 < NCs |
| Payne J M et al., 2011^[6]^ | NF1 mean age=10.62 y  NCs mean age=11.24 y | NF1=199  NCs=55 | BRIEF | WM | NF1 < NCs |
| Hyman S L et al., 2005^[7]^ | NF1 age range=8-16 y  NCs age range=8-16 y | NF1=81  NCs=49 | The Gap Matching task from BORB  The Line Orientation tasks from BORB | Visual-spatial WM  Visual-spatial WM | NF1 < NCs  NF1 < NCs |
|  |  |  | Digit Span Backward + Digit Span Forward minus Digit Span Backwards | Verbal WM | — |
| Champion J A et al., 2014^[8]^ | NF1 age range=7-17 y  NRV | NF1=46  NRV | WMI of the WISC-IV  SWM BSE of CANTAB  SWM strategy of CANTAB | WM  Spatial WM  Spatial WM | NF1 < NCs  NF1 < NCs  NF1 < NCs |
| Gilboa Y et al.2014^[9]^ | NF1 mean age=12.3 y  NCs mean age=12.4 y | NF1=29  NCs=27 | BRIEF-P | WM | NF1 < NCs |
| Ullrich N J et al., 2010^[10]^ | NF1 mean age=12.8 y  NCs mean age=12.7 y | NF1=10  NCs=6 | CANTAB | Spatial WM | NF1 < NCs |
| Huijbregts S et al., 2010^[11]^ | NF1 age range=6.9–17.4 y  NCs age range=6.0–17.3 y | NF1=30  NCs=30 | The Memory Search 2D Objects (part 1)  The Memory Search 2D Objects (part 2)  The Visuo-Spatial Sequencing-task | WM  WM  Visual-spatial WM + Visuo-temporal WM | —  NF1 < NCs  Both NF1 < NCs |
| Plasschaert E et al., 2016^[12]^ | NF1 age range=8-18 y  NCs age range=8-18 y | NF1=42  NCs=52 | CANTAB  WNV-NL | Spatial WM  Spatial WM | NF1 < NCs  NF1 < NCs |
| Rowbotham I et al., 2009^[13]^ | NF1 age range=11.9–16.8 y  NCs age range=12.2 –16.3 y | NF1=16  NCs=16 | The Memory Search 2D Objects | WM | NF1 < NCs |
| Ferner R E et al., 1996^[14]^ | NF1 age range=6-75 y  NCs age range=6-75 y | NF1=103  NCs=105 | The Sternberg Memory Task | WM | NF1 < NCs |
| Shilyansky C et al., 2010^[15]^ | NF1 mean age=24.00 y  NCs mean age=22.58 y | NF1=14  NCs=12 | Two spatial delayed response tasks | Spatial WM | NF1 < NCs |
| Descheemaeker M J et al., 2013^[16]^ | NF1 mean age=41.1 y  NCs mean age=41.8 y | NF1=20  NCs=20 | Auditory Verbal Learning Test (Dutch version) | Auditory WM | NF1 < NCs |
| Costa Dde S et al., 2014^[17]^ | NF1 mean age=65 y  NCs mean age=70 y | NF1=5  NCs=49 | Digit Span Backward + Digit Span Forward  Corsi Span Backward + Corsi Span Forward | Verbal WM  Spatial WM | NF1 < NCs  NF1 < NCs |

**S1** Previous literature involving WM in NF1 patients

—: no group difference; NF1 performed poorer than NCs; BORB: the Birmingham Object Recognition Battery; BRIEF: Behavior Rating Inventory of Executive Function; BRIEF-P: Behavior Rating Inventory of Executive Function-Preschool; CANTAB: The Spatial Working Memory subtask of the Cambridge Neuropsychological Testing Automated Battery; NCs: normal controls; LNS: the University of Maryland Letter–Number Sequencing task; mo: months; NF1: patients with neurofibromatosis type 1; NRV: normative reference values; SCAP: Spatial Capacity Working Memory Task; SWM BSE: spatial working memory between search errors; SWM strategy: spatial working memory strategy score; WISC-IV: The Wechsler Intelligence Scale for Children–Fourth Edition; WMI: The Working Memory Index; WNV-NL: The Spatial Span subtest of the Wechsler nonverbal scale of ability (Dutch version)

[1] Lorenzo J, Barton B, Arnold S S. Cognitive Features that Distinguish Preschool-Age Children with Neurofibromatosis Type 1 from Their Peers: A Matched Case-Control Study [J]. J Pediatr, 2013, 163(5): 1479-83.doi:10.1016/j.jpeds.2013.06.038

[2] Casnar C L, Klein-Tasman B P. Parent and Teacher Perspectives on Emerging Executive Functioning in Preschoolers With Neurofibromatosis Type 1: Comparison to Unaffected Children and Lab-Based Measures [J]. Journal of pediatric psychology, 2017, 42(2): 198-207.doi:10.1093/jpepsy/jsw042

[3] Chaix Y, Lauwers-Cancès V, Faure-Marie N, et al. Deficit in phonological processes: a characteristic of the neuropsychological profile of children with NF1 [J]. Child Neuropsychology, 2017, 24(4): 558-74.doi:10.1080/09297049.2017.1313970

[4] Payne J M, Arnold S S, Pride N A, et al. Does attention-deficit-hyperactivity disorder exacerbate executive dysfunction in children with neurofibromatosis type 1? [J]. Dev Med Child Neurol, 2012, 54(10): 898-904.doi:10.1111/j.1469-8749.2012.04357

[5] Lehtonen A, Garg S, Roberts S A, et al. Cognition in children with neurofibromatosis type 1: Data from a population-based study [J]. Dev Med Child Neurol, 2015, 57(7): 645-51.doi:10.1111/dmcn.12734

[6] Payne J M, Hyman S L, Shores E A, et al. Assessment of executive function and attention in children with neurofibromatosis type 1: relationships between cognitive measures and real-world behavior [J]. Child Neuropsychol, 2011, 17(4): 313-29.doi:10.1080/09297049.2010.542746

[7] Hyman S L, Shores A, North K N. The nature and frequency of cognitive deficits in children with neurofibromatosis type 1 [J]. Neurology, 2005, 65(7): 1037-44.doi:10.1212/01.wnl.0000179303.72345.ce

[8] Champion J A, Rose K J, Payne J M, et al. Relationship between cognitive dysfunction, gait, and motor impairment in children and adolescents with neurofibromatosis type 1 [J]. Dev Med Child Neurol, 2014, 56(5): 468-74.doi:10.1111/dmcn.12361

[9] Gilboa Y, Rosenblum S, Fattal-Valevski A, et al. Is there a relationship between executive functions and academic success in children with neurofibromatosis type 1? [J]. Neuropsychological rehabilitation, 2014, 24(6): 918-35.doi:10.1080/09602011.2014.920262

[10] Ullrich N J, Ayr L, Leaffer E, et al. Pilot Study of a Novel Computerized Task to Assess Spatial Learning in Children and Adolescents With Neurofibromatosis Type 1 [J]. Journal of Child Neurology, 2010, 25(10): 1195-202.doi:10.1177/0883073809358454

[11] Huijbregts S, Swaab H, de Sonneville L. Cognitive and motor control in neurofibromatosis type I: influence of maturation and hyperactivity-inattention [J]. Dev Neuropsychol, 2010, 35(6): 737-51.doi:10.1080/87565641.2010.508670

[12] Plasschaert E, Van Eylen L, Descheemaeker M J, et al. Executive functioning deficits in children with neurofibromatosis type 1: The influence of intellectual and social functioning [J]. Am J Med Genet B Neuropsychiatr Genet, 2016, 171B(3): 348-62.doi:10.1002/ajmg.b.32414

[13] Rowbotham I, Pit-ten Cate I M, Sonuga-Barke E J, et al. Cognitive control in adolescents with neurofibromatosis type 1 [J]. Neuropsychology, 2009, 23(1): 50-60.doi:10.1037/a0013927

[14] Ferner R E, Hughes R A, Weinman J. Intellectual impairment in neurofibromatosis 1 [J]. J Neurol Sci, 1996, 138(1-2): 125-33.doi:10.1016/0022-510x(96)00022-6

[15] Shilyansky C, Karlsgodt K H, Cummings D M, et al. Neurofibromin regulates corticostriatal inhibitory networks during working memory performance [J]. Proceedings of the National Academy of Sciences, 2010, 107(29): 13141-6.doi:10.1073/pnas.1004829107

[16] Descheemaeker M J, Plasschaert E, Frijns J P, et al. Neuropsychological profile in adults with neurofibromatosis type 1 compared to a control group [J]. J Intellect Disabil Res, 2013, 57(9): 874-86.doi:10.1111/j.1365-2788.2012.01648.x

[17] Costa Dde S, de Paula J J, de Rezende N A, et al. Neuropsychological impairments in elderly Neurofibromatosis type 1 patients [J]. Eur J Med Genet, 2014, 57(5): 216-9.doi:10.1016/j.ejmg.2014.02.004
